# Probing Active Sites and Reaction Intermediates of Electrocatalysis Through Confocal Near-Infrared Photoluminescence Spectroscopy: A Perspective

**DOI:** 10.3389/fchem.2020.00327

**Published:** 2020-04-28

**Authors:** Vidhya Chakrapani

**Affiliations:** ^1^Howard P. Isermann Department of Chemical and Biological Engineering, Rensselaer Polytechnic Institute, Troy, NY, United States; ^2^Department of Physics, Applied Physics, and Astronomy, Rensselaer Polytechnic Institute, Troy, NY, United States

**Keywords:** electrocatalysis, OER, oxidation state, photoluminescence spectroscopy (PL), intermediates and mechanism, X-ray photoemission spectroscopy (XPS), X-ray absorption spectroscopy (XAS)

## Abstract

Electrocatalytic reactions such as oxygen evolution (OER) and oxygen reduction reactions (ORR) are one of the most complex heterogeneous charge transfer processes because of the involvement of multiple proton-coupled-electron transfer steps over a narrow potential range and the formation/breaking of oxygen-oxygen bonds. Obtaining a clear mechanistic picture of these reactions on some highly active strongly-correlated oxides such as MnO_x_, NiO_x_, and IrO_x_ has been challenging due to the inherent limitations of the common spectroscopic tools used for probing the reactive intermediates and active sites. This perspective article briefly summarizes some of the key challenges encountered in such probes and describes some of unique advantages of confocal near-infrared photoluminescence (NIR-PL) technique for probing surface and bulk metal cation states under *in-situ* and *ex-situ* electrochemical polarization studies. Use of this technique opens up a new avenue for studying changes in the electronic structure of metal oxides occurring as a result of perturbation of defect equilibria, which is crucial in a broad range of heterogeneous systems such as catalysis, photocatalysis, mineral redox chemistry, and batteries.

## Introduction

Electrocatalytic oxygen evolution and oxygen reduction reactions underpin the efficient operation of many reversible electrochemical energy conversion and storage devices such as solar cells, regenerative fuel cells (Adler, 2004; Gasteiger et al., 2005), and rechargeable metal-air batteries (Cao et al., 2012) that have theoretical energy densities (10–12 kWh/kg) on par with the energy density of gasoline (~13 kWh/kg) (Yang et al., 2011). Consequently, considerable efforts have been dedicated toward improving the energy conversion efficiency of these devices (Badwal et al., 2014; Yoo et al., 2014). However, the main obstacle for achieving this has been the low activity of electrocatalysts employed for the ORR and OER reactions that occur during charge/discharge cycles (Chen et al., 2002). Oxygen reactions are known to be complex charge transfer processes because they involve multiple proton-coupled electron transfer. Electrocatalysts that can efficiently catalyze these reactions are either limited or unstable, which leads to energy losses and decreased lifetime of systems employing them. Some of the best known electrocatalysts for oxygen reactions are Pt, RuO_2_, and IrO_2_, but their high cost and low availability is an issue (Horkans and Shafer, 1977; Maiyalagan et al., 2014; Hosseini-Benhangi et al., 2015).

Currently, there is a great interest in the study of Mn-based electrocatalysts for OER/ORR processes. The multi-electron OER process that occurs in nature as a result of photosynthetic water oxidation by green plants, algae, and cyanobacteria has been shown to be catalyzed by a Mn_4_CaO_5_ cluster located inside the membrane protein complex called photosystem II (PS-II) (Cox et al., 2014) at a record low overpotential of 160–300 mV at pH = 5.5 with an incident-light-to-fuel conversion efficiency of 16% and turn over frequency (TOF) of 1,000 s^−1^ (Dau and Zaharieva, 2009; Dau et al., 2010). For comparison, the best benchmarked IrO_x_ and RuO_2_-based OER catalysts have overpotentials between 300 and 400 mV depending on the pH conditions with a TOF of 0.1–1 s^−1^ (Hong et al., 2015). One reason for the low TOF of the artificial catalysts compared to the TOF of Mn_4_CaO_5_ of PS-II has been postulated to be related to the efficiency of the charge accumulation process, i.e., the ease of formation of higher-valent metal cation states that can stabilize the higher-order oxygen intermediates. Among the first-row transition metals, element Mn has the highest accessible stable valency, which is +7, and also has the largest range of stable oxidation states (Mn^2+^ to Mn^7+^). This ability of Mn to support at least five charges is likely why nature chose Mn as the preferred element for the OER process, which requires accumulation of four charges to convert two water molecules to molecular oxygen. IrO_x_, one of the most active OER catalysts in both acidic and basic electrolytes, is also believed to oxidize H_2_O through a high-valent Ir^5+^ intermediate, although it has not yet been conclusively proved. Thus, a knowledge of the nature and charge state of reactive intermediates can help better understand the differences between synthetic and natural electrocatalysts and in turn aid in their better design.

Understanding the mechanism of electrocatalytic reactions, such as water oxidation, requires the knowledge of both the type of reactive intermediates as well as the nature of active sites that enable adsorptive binding. This crucially depends on our ability to characterize the electrocatalyst in their working state. However, no single experimental probe can entirely capture the complexity of such processes, which involves multiple one-electron steps. Instead, the information from multiple probes have to be correlated in order to obtain a global picture.

Experimental techniques to measure metal oxidation states are limited. Common techniques include X-ray absorption (XAS) and photoemission (XPS) spectroscopy (Adarsh et al., 2019). While being extremely valuable for providing structural information, they provide information on the average oxidation state only when used for studying strongly-correlated oxides such as NiO_x_, and MnO_x_, while it is known that the working catalyst under polarization is multi-valent (Silvester et al., 1997; Iuzzolino et al., 1998). Similarly, electron paramagnetic resonance (EPR), another powerful technique, only probes paramagnetic metal oxidation states (Iuzzolino et al., 1998; McAlpin et al., 2010). This limitation in probing the dynamic range of the accessible metal valence states especially near the potential of OER/ORR has been the bottleneck in the precise determination of the electronic state of the catalytically active site and its correlation to the electrochemical activity both in PS-II as well as in other synthetic systems.

This perspective article briefly summarizes the benefits of some of the useful spectroscopic tools available for probing valence states, active sites, and reactive intermediates of OER/ORR as well as the limitations of these techniques. In particular, this article elucidates the salient benefits of confocal near-infrared photoluminescence spectroscopy as a relatively novel probe for the use in *ex-situ* and *in-situ* electrocatalytic studies and summarizes some of our recent results.

## Background

### Mechanism of OER/ORR-Nature of Reaction Intermediates

It has now been well-established that OER and ORR on metal oxides proceed via formation of a series of reaction intermediates that involves the binding of the electrolyte ions (typically OH^−^, H^+^) and H_2_O molecules to the metal and oxygen (${\text{O}}_{2}^{-}$) lattice sites that serve as the catalytic active center for the reaction. For instance, in alkaline environments, OER can be written as

(1) $$\begin{array}{l} 4OH^{-}\to 2H_{2}O+4e^{-}+O_{2}↑ \end{array}$$

**Figure 1:**
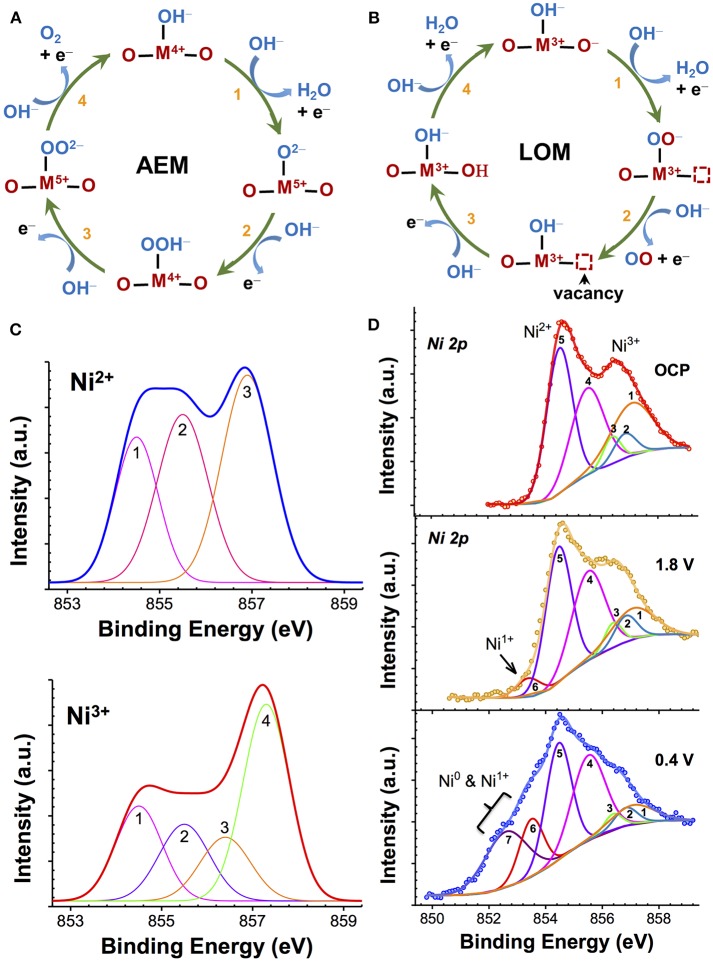
**(A, B)** Two types of OER mechanisms seen in transition metal oxides. In adsorbate evolution mechanism (AEM), metal cation site (M) is the active site, and undergoes an increase in the oxidation state **(A)**, while in lattice-oxygen mechanism (LOM) a lattice oxygen site is the active site that undergoes redox changes with the resulting formation of a vacancy defect **(B)**; **(C)** Theoretically-calculated multiplet envelopes of Gupta and Sen (1974, 1975) and McIntyre and coworkers (Grosvenor et al., 2006) for free Ni^2+^ and Ni^3+^ ions; and **(D)** XPS spectra of NiO_x_ showing Ni 2p_3/2_ core level before (OCP) and after Li^+^ insertion at potential of 1.8 V and 0.4 V vs. Li/Li^+^ fitted with Ni^2+^ and Ni^3+^ multiplet envelopes, as reported in Wang et al. (2016b).

The reverse of this reaction is ORR. The overall reaction, as given by Reaction 1, is generally shown to occur as a series of one-electron intermediates (Bockris and Otagawa, 1983; Goodenough et al., 1990; Dau et al., 2010; Man et al., 2011; McCrory et al., 2013; Mavros et al., 2014; Hong et al., 2015). When the intermediates involve the sequential formation of ^*^OH, ^*^O, ^*^OOH, and ^*^OO (Figure 1A) at the metal cation site (^*^, active site), which undergoes an increase in the oxidation state, the mechanism is commonly known as the adsorbate evolution mechanism (AEM). This is the preferred mechanism for stable catalysis and generally results in moderate activity (Goodenough et al., 1990; Man et al., 2011; Chen et al., 2017). Experimental studies on active binary oxide catalysts such as IrO_x_, MnO_x_, and NiO_x_ have shown that these catalysts show an increase in the average oxidation state (>3) at potentials before OER, which is considered to be the key step for efficient AEM-OER catalysis, while a decrease in the oxidation state (<3) during ORR (Kötz et al., 1983; Augustynski et al., 1984; Kötz and Neff, 1985; Nahor et al., 1991; Mo et al., 2002; Gorlin et al., 2013; Risch et al., 2017). In contrast, if the OER process involves the participation (abstraction) of lattice oxygen atoms that occurs in more covalent oxides [RuO_2_ (Stoerzinger et al., 2017), many perovskites], which was first proposed by Matsumoto et al. (1977, 1980) and Matsumoto and Sato (1986) then the mechanism is referred to as lattice oxygen-mechanism (LOM) (Matsumoto and Sato, 1986; Mefford et al., 2016). In this reaction scheme (Figure 1B), the oxidation state of the lattice oxygen atoms changes while the metal redox state remains nearly constant. Therefore, identification of the cation oxidation state can provide an insight into the nature of OER/ORR process. Although many different routes can exist (Bockris and Otagawa, 1983; Man et al., 2011; Mavros et al., 2014), as illustrated neatly in recent review articles (Dau et al., 2010; McCrory et al., 2013; Hong et al., 2015) one of the more commonly evoked reaction schemes in basic electrolytes is shown in Figures 1A,B. The key parameter that determines the type of OER process is the hydroxide affinity of the semiconductor/electrolyte interface. Many highly covalent oxides that have a large work function (Hong et al., 2017) exhibit negative hydroxide affinity due to the presence of an e^−^ accumulation at the surface in aqueous electrolyte, which repels the negative OH^−^ from the solid/electrolyte interface. This OH^−^ deficiency at the interface results in insufficient ionic compensation in the oxide during polarization. As a result, the localization of holes at the lattice oxygen site leads to the formation of O^−^ intermediate and a subsequent generation of oxygen vacancy (V_O_) during OER catalysis (LOM pathway) (Rong et al., 2016; Grimaud et al., 2017). Many in-depth studies on perovskites by Bockris and Otagawa (1983, 1984, 2002), Matsumoto et al. (1980), and Matsumoto and Sato (1986) have pointed out the instability of such perovskites. Consequently, these oxides, while being highly efficient at OER (low Tafel slopes), are unstable in bulk and result in cation leaching and surface amorphization (Lee et al., 2011; May et al., 2012; Chang et al., 2014; Forslund et al., 2018). In contrast, stable AEM materials show positive hydroxide affinity that promotes insertion/adsorption of OH^−^ lattice during OER and therefore supports the subsequent formation of higher-order O intermediates (=O, OOH, OO).

In addition, pH has a strong effect on the nature of adsorbate intermediates as well as on the charge on the metal cation. The nature of adsorbate intermediates depends strongly on the pH of the electrolyte because the surface charge caused by protonation/deprotonation of surface oxygen-functional species depends on the pH of zero charge (pzc) of the oxide surface relative to the solution pH (Noh and Schwarz, 1989; Pechenyuk, 1999). At pH values higher than the pzc, negatively-charged species accumulate easily on the surface (OH^−^, OOH^−^, O^2−^). Therefore, a lower overpotential is expected for OER in basic solutions than those at acidic/neutral pH (Goodenough et al., 1990; Goodenough and Cushing, 2003; Takashima et al., 2012b) pH can also affect the stability of the metal cation active sites and may lead to their charge disproportionation. pH-dependent studies on δ-MnO_2_ have shown that the Mn^3+^ defect site, which is the proposed active site for OER, is unstable at pH <9 and undergoes disproportionation to Mn^2+^ and Mn^4+^ defects, whereas Mn^3+^ is effectively stabilized by the comproportionation of Mn^2+^ and Mn^4+^ at higher pH conditions (Takashima et al., 2012a,b).

All-in-all, probing the changes in the anion and cation oxidation states at various potentials preceding OER/ORR steps is necessary for identifying the nature of the active site, type of reactive intermediates, and stability of metal cations, which in turn would help in understanding the overall mechanism of the complex OER/ORR processes.

### Common Techniques for Probing Active Sites/Intermediates and Their Limitations

Manganese can exist in oxidation states from −3 to +7, and as a result form many strongly-correlated phases of manganese oxides (MnO_x_) of more than 30 different polymorphs (Post, 1999; Meng et al., 2014). This makes Mn one of the most complex elements to study. Common techniques for determining the oxidation state of the catalysts are XPS, XAS, EPR, and absorption (UV-Vis) measurements.

#### X-ray Photoemission Spectroscopy (XPS)

XPS is one of the most common and powerful techniques available today for obtaining both electronic and compositional information of electrocatalysts. Earliest studies employed surface-sensitive XPS on “emersed” electrodes that were polarized *ex-situ* at different potentials. Pioneering studies of Kolb, Kötz, Neff, and Ross on emersed electrodes have shown that the electrical double layer of the polarized electrode is preserved upon their emersion and the subsequent transfer into an ultrahigh vacuum (UHV) chamber, and therefore can be used for detailed surface-structural studies through X-ray and ultraviolet photoemission studies (Hansen et al., 1980; Rath and Kolb, 1981; Kolb et al., 1983; Kötz et al., 1983, 1986; Wagner and Ross, 1983; Kötz and Neff, 1985). Electrochemical studies on IrO_x_ show that the oxidation state of the metal cation increases with anodic polarization preceding the OER step (Goodenough et al., 1990). On the other hand, results of XPS studies of Kötz et al. on emersed IrO_x_ have not been conclusive. While an increase in the intensity of Ir 4f on higher BE was observed, the authors concluded that a definite correlation of the Ir 4f binding energy (BE) with an Ir oxidation state cannot be made from measured data (Kotz et al., 1984; Kötz and Neff, 1985). From the analysis of the O 1s signal at various anodic potentials, they showed that the contribution of hydroxide species to the overall O 1s signal decreases with anodic polarization.

In the recent decade, advancements in ambient-pressure XPS (APXPS) have enabled in-operando studies of the electrical double layer (Favaro et al., 2016) and have led to the identification of some of the keys intermediates of water oxidation (Yamamoto et al., 2008; Sanchez Casalongue et al., 2014). Further, the use of different X-ray energies has allowed for surface and bulk contributions to be differentiated through variation in the probing depth. *In-situ* studies of Nilsson and coworkers (Sanchez Casalongue et al., 2014) on IrO_x_ show that the intensity of Ir 4f line increases at higher binding energy (BE) upon polarization at OER potentials along with a decrease in surface hydroxide concentration, which is similar to the results of Kotz et al. (1984) on emersed IrO_x_ electrode. The increase in the high BE signal was attributed to the formation of Ir^5+^ at the surface and was taken to be evidence for OOH–mediated OER mechanism. In contrast, the comparative XPS studies of amorphous and crystalline IrO_x_ by Pfeifer et al. (2016) indicate that the increased activity of amorphous oxide is due to the presence of both anionic (O^−^) and cationic (Ir^3+^) defects in the lattice, which casts doubt on the OOH-mediated OER mechanism in active IrO_x_.

##### Limitations when probing correlated-oxides

The determination of the relative contribution of different metal oxidation states (such as Mn^2+^, Mn^3+^, and Mn^4+^) through XPS in strongly-correlated oxides such as MnO_x_, IrO_x_, or NiO_x_ is highly complex and challenging because of the overlapping cationic signals (Oswald and Brückner, 2004; Grosvenor et al., 2006; Biesinger et al., 2009). The photoionization process resulting from the X-ray absorption results in the creation of vacancies (holes) in the inner core levels. The strong spin-orbit and electrostatic interactions between the vacancies and the unpaired valence-shell (d) electrons of high spin cation in strongly-correlated systems such as Mn, Fe, and Ni, causes a severe broadening of energy of the photoelectrons (Pal and Gupta, 1982). In a multi-valent sample, the overlapping of broadened peaks of various oxidation state cations makes the assignment of oxidation state to a single identifiable peak binding energy (BE) very difficult. This is demonstrated in Figures 1C,D for NiO_x_. In such cases, the individual contribution of different metal cations is generally resolved by fitting the main emission peak using theoretically-calculated multiplet envelopes of free ions, such as that predicted by Gupta and Sen (1974, 1975) and successfully applied by Grosvenor et al. (2004, 2006) and elaborated further by Nesbitt and Banerjee (1998) for various MnO_x_ and by us (Wang et al., 2016b) for NiO_x_. For instance, the multiplet envelope of free Ni^2+^ and Ni^3+^ ions, as shown in Figure 1C, are used for XPS fitting (Figure 1D) of a multi-valent NiO_x_ containing Ni^3+^, Ni^2+^, Ni^+^, and Ni^0^ that was obtained with Li^+^ ion insertion into stoichiometric NiO_x_ (Wang et al., 2016b). Such a method is highly prone to error when changes in the relative signal intensity of different metal cations with electrochemical polarization under OER must be measured.

In addition to the uncertainty in the assignment of oxidation state to a single defined BE position, XPS studies by Pfeifer et al. (2016) on highly active amorphous IrO_x_ electrocatalyst show that the presence of lower-valent Ir^3+^ species in predominately Ir^4+^ cationic framework lead to a reverse BE shift, i.e., it causes an increase in signal intensity on the higher BE side of the Ir 4f line as opposed to the expected lower BE side. These results highlight the need for caution when assigning oxidation states solely based on BE shifts in XPS. Such reverse BE shifts have also been reported in oxides such as AgO_x_, which has been attributed to the screening of the core-hole by metal s states (Gaarenstroom and Winograd, 1977; Kaspar et al., 2010; Grönbeck et al., 2012).

#### X-ray Absorption Spectroscopy (XAS) measurements

Oxidation state changes of the catalysts have also been monitored through relatively more bulk-sensitive techniques such as X-ray absorption near-edge (XANES) and extended X-ray absorption fine structure (EXAFS). XAS is an atom-specific probe for both the local geometry of the metal cation as well as its valence state. It can provide important information not only on the valence states but also the interatomic distance, the coordination number and the spread of distances in the first coordination shell. In addition, XAS, unlike XPS measurements, can probe the electrocatalyst under in-operando working conditions in the presence of electrolyte under various polarizations. XAS being a local probe is also advantageous over the X-ray diffraction technique, which cannot resolve crystallites smaller than 5 nm due to the broadening of Bragg peaks. However, XAS is capable of resolving both bulk and nanocrystalline (or amorphous) phases.

*In-situ* XAS measurements on IrO_x_ electrocatalysts have shown that the average Ir valence increases from 3 at OCP up to 4.8 at the potentials of OER (Hüppauff and Lengeler, 1993; Mo et al., 2002); the non-integer value of valence indicates that the electrocatalyst likely contain mixtures of Ir^3+^, Ir^4+^, and Ir^5+^ states. Similarly, several in-depth characterization studies of various MnO_x_ phases under electrochemical polarizations through XAS measurements have shown that the average oxidation state of Mn increases during OER and decreases to during ORR, as seen from the shifts of the absorption edge to higher or lower energy (Silvester et al., 1997; Gorlin et al., 2013; Risch et al., 2017). Like in XPS, interactions between core holes and valence electrons also results in the broadening of Mn K edge lines in the XAS measurements, which result in the loss of sensitivity and makes resolution of individual chemical states in mixed valent oxide difficult (Manceau et al., 1992). Another disadvantage of XAS is that the technique relies on the calibration of a catalyst spectrum with a spectrum of a reference oxide sample, which in itself may be of mixed valence, as most pure MnO_x_ phases are seldom perfectly stoichiometric. As a result, the nature of active sites for OER on such sites has not been identified with certainty.

## Near-Infrared Photoluminescence Spectroscopy

In contrast to XAS and XPS measurements, photoexcitation during PL measurements for different metal cations can be done using a relatively low energy laser and therefore only involves excitation of valence electrons to the higher energy state without core-level excitations. Hence, peak-broadening that limits precise identification of cationic states is not an issue in PL.

PL spectroscopy relies on the detection of the photons emitted from a material when light of sufficient energy is incident upon it. The absorption of the incident photon results in the excitation of electrons from the ground states to higher energy states and their subsequent relaxation back to the ground state by the release of excess energy. When the excess energy is released in the form of photons (radiative transitions), then the process is called photoluminescence. On the other hand, a part of this excess energy from electron transitions to lower energy states also occurs through the release heat or phonons, which is a non-radiative process. Therefore, the intensity of PL spectrum (or quantum efficiency) gives a measure of the relative rates of radiative to non-radiative recombination process. In general, the PL spectrum provides a wealth of information about the electronic structure of the material. The energy of the peak positions denotes the energy difference between the electronic states between which the electron-hole recombination occurs, and therefore can provide information regarding the electronic bands, doping, bulk, and interface trap levels (Gfroerer, 2006). It is a selective and extremely sensitive probe of discrete electronic states. In addition, it is a non-destructive technique that requires very little sample preparation and most importantly, does not require any UHV environment. It can be used to study both electrically conducting and non-conducting phases.

The main drawback of the PL technique is that it can only detect radiative emissions. Therefore, information about non-radiative transitions such as that originating from traps or recombination centers must be obtained indirectly. As a result, materials such as amorphous semiconductors, low-crystal quality films, or indirect band gap semiconductors that have low quantum efficiency due to high e^−^-h+ recombination occurring via non-radiative pathways are difficult to study through commonly used PL instruments. Furthermore, while the intensity of a PL peak is proportional to the density of the radiative states, unlike absorption measurements, the determination of their absolute density (concentration) is not straightforward.

**Figure 2:**
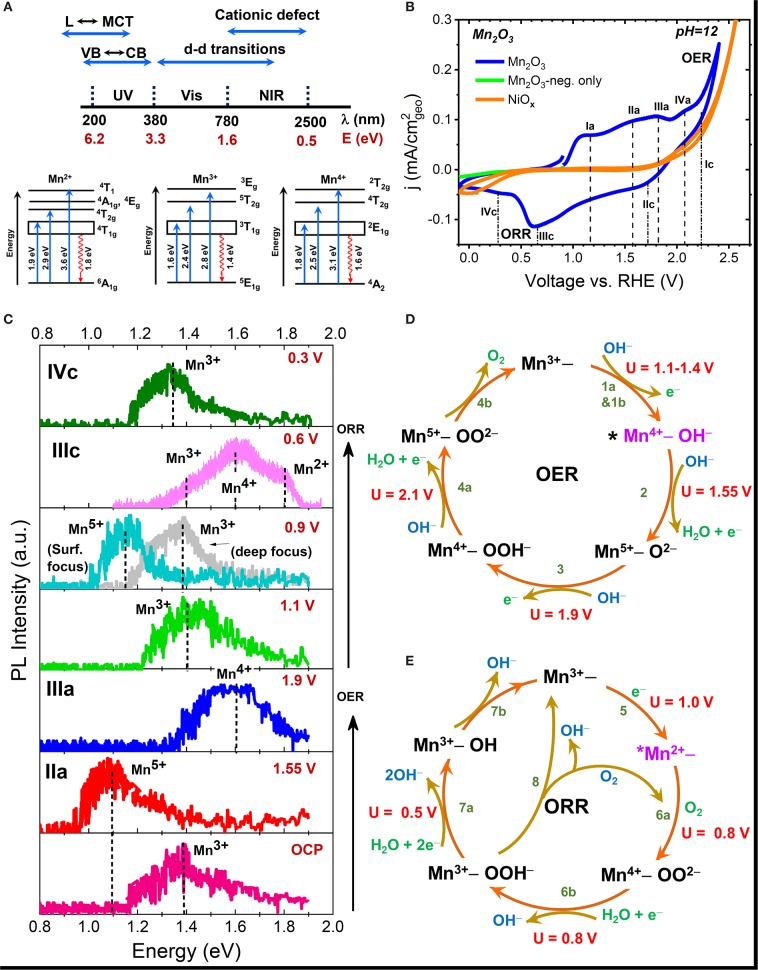
**(A)** Schematic showing the spectral energy range of various radiative transitions seen in metal oxides along with the various electronic transitions of Mn^2+^, Mn^3+^, and Mn^4+^ cations; **(B)** Cyclic voltammograms of Mn_2_O_3_ and NiO_x_ electrodes obtained in pH = 12 aqueous KOH electrolyte; **(C)** *Ex-situ* NIR PL spectra of emersed Mn_2_O_3_ electrode after polarization in pH = 12 electrolyte at various peak potential seen in CV. The observed surface shifts in the emission peak reflects the changes in the oxidation state of Mn atom during polarization; and **(D,E)** Schematic of the mechanistic pathway of the OER **(D)** and ORR **(E)** processes on Mn_2_O_3_ showing the potential (U)-dependent formation of various intermediates. The * in the figure represents the active site, which in Mn_2_O_3_ was deduced to be Mn^4+^ defect site for OER and Mn^2+^ site for ORR. Data taken from Roy et al. (2020).

The spectral range of radiative emissions seen in transition metal oxides (TMOs) is shown in Figure 2A (Sherman, 1984). Many TMOs are strongly ionic, and hence have band-to-band or excitonic emissions that lie in the ultraviolet (UV) region. Electronic transitions between ligand (L) to metal charge transfer (MCT) and metal to conduction band (CB) transitions frequently occur in the UV spectral range. The d-d transitions are usually the visible region, and defect-related emissions, both cationic and anionic, in the visible to NIR spectral region. In NiO_x_, the Ni^+1^, Ni^+2^, and Ni^+3^ have characteristic emission in the 0.7–0.9 eV, while in MnO_x_, the characteristic emission of various Mn cationic defects (Mn^2+^-Mn^6+^) occur in the 0.9–1.9 eV spectral range (Mićić and Draškovi, 1985; Sekiguchi and Adachi, 2015; Silva et al., 2016; Ren and Yang, 2018). Thus, monitoring the changes in emission in the NIR range can provide direct information regarding changes in the oxidation state of the metal cation that occur as a result of electrochemical polarization.

Ultraviolet to visible range PL technique has been successfully used to study high-temperature heterogeneous catalysts. Seminal work in the field was done by Anpo et al., who used visible PL for catalytic studies on metal oxides, and showed the applicability of this technique for obtaining real-time information about various surface chemical phenomena, such as changes in the structure, coordination, and reactivity of metal centers that occur as a result of adsorption of species during catalysis (Anpo et al., 1985, 1988; Anpo and Che, 1999; Matsuoka et al., 2008).

We recently reported through studies (Puntambekar et al., 2016; Wang et al., 2016a,b) on several different oxides and chalcogenides the first use of NIR-PL as a technique for studying various cationic defects under *in-operando* and *ex-situ* electrochemical conditions. PL/Raman spectroscopy is a fairly routine technique that is more easily accessible than the synchrotron X-ray facility. Other advantages are that it can be highly surface-sensitive, it is capable of detecting radiative emission from defects at concentrations as low as 10^18^ cm^−3^, and with certain precautions can be easily adapted for *in-situ* catalytic studies that may involve the presence of a metal catalyst (<5 wt%). Without requiring any setup change, the instrument can also obtain Raman spectra. Vibration spectroscopy such as Raman scattering is one of the most sensitive techniques available for structural characterization because of its ability to probe amorphous phases and those with short-range order, such as the highly disordered phases of MnO_x_. The technique can be directly used to probe the near-neighbor environment of the oxygen coordination around Mn and alkali cation (Julien et al., 2003). Thus, the combined NIR-PL/Raman measurement is uniquely suited to obtain simultaneous electronic and structural information of the catalyst. The spatial resolution of PL/Raman system is usually in micrometers, and therefore cannot provide information on spatial distribution of defects at the nanoscale.

### Distinguishing Surface vs. Bulk States Through Confocal Microscopy

The penetration depth of the excitation light, which is a measure of thickness of material that is probed, in any material depends strongly on the energy of the incident light and is given by the inverse of the wavelength-dependent absorption coefficient. The surface sensitivity of the PL technique depends both on the wavelength of light used as well as on the electronic structure of the material. When direct band gap semiconductors are probed using UV or an above-band gap energy excitation source, the penetration depth is small, spanning tens to hundreds of nanometers. While the diffusion of photoexcited charge carrier would tend to increase the probe depth, the PL spectrum in such a case is quite sensitive to surface effects and recombination. In contrast, penetration depth can span several tens of microns when absorption is weaker as in the case of indirect band gap semiconductors or when excitation is done using a sub-band gap energy light source. Here, PL signal is dominated by bulk recombination processes. Therefore, spectra obtained using multiple excitation wavelengths can help distinguish surface vs. bulk states. Another strategy is to utilize a confocal PL microscope with a three-dimensional (3D) optical resolution capability. Confocal microscopes work on the principle of point excitation in the specimen (diffraction limited spot) and point detection of the resulting fluorescence signal. In most confocal setups, 3D resolution is achieved through the use of a pinhole placed in front of the detector such that signal originating from an in-focus plane passes freely through the pinhole and is imaged by the detector, whereas light coming from out-of-focus planes is physically blocked from passing through the pinhole and reaching the detector. Raster scanning the specimen one point at a time permits thin optical sections to be collected by simply changing the z-focus. The resulting images can be stacked to produce a 3D fluorescence image of the catalysts. In this way, one can obtain depth-dependent changes in the PL spectrum that can be used to distinguish bulk vs. surface intermediates without sample destruction or movement.

### *Ex-situ* Electrocatalytic Studies of OER/ORR on Manganese Oxides

In our recent work (Roy et al., 2020), we demonstrated the successful application of *ex-situ* NIR-PL technique to track the OER and ORR intermediates and identify the nature of catalytic sites on the most active MnO_x_ phase, namely Mn_2_O_3_. Mn_2_O_3_ is one of the very few bifunctional electrocatalyst capable of catalyzing both OER and ORR processes. Such catalysts are of tremendous importance in renewable electrochemical devices such as metal-air batteries, regenerative fuel cells, and electrolyzers.

Successful application of NIR-PL for the detection of reaction intermediates on a wide variety of electrocatalysts requires development of a database of characteristic emission energies of different metal cations. For the case of Mn cation, reference spectra obtained with mineral samples (or binary oxides) of known composition gives the characteristic peak emissions of various oxidation states: Mn^2+^ at 1.8 eV, Mn^3+^ at 1.35 eV, Mn^4+^ at 1.6 eV, and Mn^5+^ at 1.1 eV, and are consistent with the values reported in literature (Sherman, 1984; Hozoi, 2003; Xiao et al., 2017; Cao et al., 2018). Readers can refer to the supporting information of (Roy et al., 2020) for further details. Figure 2A shows the schematic of electronic transitions giving rise to the characteristic absorption and emission signals (Hozoi, 2003) of various Mn cations. Figure 2B shows the cyclic voltammogram of a Mn_2_O_3_ electrode in Ar-purged electrolyte along with the labels for all the various potentials of peak currents. Oxygen evolution was observed at potentials more positive than 1.9 V vs. reversible hydrogen electrode (RHE). The reduction of oxygen evolved during the anodic scan can be seen at potentials more negative than 0.7 V. Very little current is observed when the electrode is polarized in the cathodic direction alone. Figure 2C shows the results of *ex-situ* NIR-PL of the same electrode after polarization at various peak potentials during a linear potential scan from OCP to OER potential and then from OER potential to ORR potential in pH = 12 KOH electrolyte. All spectra were recorded using a 633 nm laser light focused on the sample surface through a confocal microscope at very low laser power to avoid spot damage due to localized laser heating. PL results confirm that the peaks seen in the voltammograms during the anodic scan correspond to the formation of Mn^4+^, Mn^5+^ oxidation states while a decrease in the oxidation state to Mn^3+^ and Mn^2+^ is observed at cathodic ORR potentials. More detailed studies in combination with gravimetric and *in-situ* absorption measurements revealed Mn^2+^ as the dominant active site for the ORR process. Measurements of spectra under different z-focus (Figure 2C) revealed that the observed formation of different Mn valence states is restricted to the surface in Mn_2_O_3_.

While our results are generally in agreement with the trend seen in XAS measurements (Risch et al., 2017), we also distinctly observed co-existing valence states together with the observation of Mn^5+^ as an intermediate of OER during electrocatalysis, as predicted by theoretical calculations (Fernando et al., 2016). The observation of potential-dependent formation of various metal charged states enabled us to map the mechanistic pathways of both OER and ORR processes (Figures 2D,E), including the identification of the active sites (Roy et al., 2020). This is shown schematically in Figure 2D for the OER process on the Mn^4+^ active site, and in Figure 2E for the ORR process on the Mn^2+^ active site. The knowledge was used to develop a band structure framework that correlates electrochemical activity with the formation energy of various metal cation intermediates. Nakamura et al. attributed one reason for the lower electrocatalytic OER efficiency of MnO_2_ and Fe_2_O_3_ catalysts compared to IrO_x_ to the charge disproportionation of high-valent states into lower-valent cations in the former oxides during OER, in contrast to the stabilization of higher-valent (Ir^5+^) state in the latter oxide (Ooka et al., 2017). Our results show for the first time that Mn_2_O_3_, which is the most active OER/ORR catalyst within the MnO_x_ system, also catalyzes OER through a high-valent Mn^5+^ intermediate. However, comparison of electrocatalytic activity rates indicate that Mn_2_O_3_ is still less active than IrO_x_. In this regard, time-dependent PL studies on the relative formation of higher valent states can shed light on the kinetics of high-valent intermediate formation and is currently underway in our group.

In summary, one bottleneck for identifying the key intermediate metal cation states using X-ray based absorption and photoemission techniques is the broadening and overlapping of the individual peaks of various metal cations due to the interactions of core hole and d electrons that makes it challenging to resolve the individual valence states in the multivalent electrocatalyst of strongly-correlated oxides. It is shown here that confocal NIR-PL spectroscopy is uniquely suited to study multi-valent states of correlated oxides under *in-situ* and *ex-situ* electrochemical conditions. In addition, the technique can also resolve bulk vs. surface active sites with three-dimensional optical resolution through a confocal microscope setup. When coupled with Raman technique, it can be a powerful tool for obtaining simultaneous structural and electronic changes occurring in the electrocatalyst in the working state.

## Data Availability Statement

All datasets generated for this study are included in the article/supplementary files.

## Author Contributions

The author confirms being the sole contributor of this work and has approved it for publication.

## Conflict of Interest

The author declares that the research was conducted in the absence of any commercial or financial relationships that could be construed as a potential conflict of interest.
